# A Safe-by-Design Approach for the Synthesis of a Novel Cross-Linked Hyaluronic Acid with Improved Biological and Physical Properties

**DOI:** 10.3390/ph16030431

**Published:** 2023-03-11

**Authors:** Sabrina Sciabica, Riccardo Barbari, Riccardo Fontana, Giovanni Tafuro, Alessandra Semenzato, Daniela Traini, Dina M. Silva, Larissa Gomes Dos Reis, Luisa Canilli, Massimo Terno, Peggy Marconi, Anna Baldisserotto, Silvia Vertuani, Stefano Manfredini

**Affiliations:** 1 Department of Life Sciences and Biotechnology, University of Ferrara, via L. Borsari 46, 44121 Ferrara, Italy; 2 Department of Chemical, Pharmaceutical and Agricultural Sciences, University of Ferrara, via Fossato di Mortara 64/B, 44121 Ferrara, Italy; 3 Unired Srl, via Niccolò Tommaseo 69, 35131 Padova, Italy; 4 Department of Pharmaceutical and Pharmacological Sciences, University of Padova, via Marzolo 5, 35131 Padova, Italy; 5 Macquarie Medical School, Faculty of Medicine, Health & Human Sciences, Macquarie University, Campus Macquarie Park, Sydney 2109, Australia; 6 Woolcock Institute of Medical Research, 431 Glebe Point Road, Glebe, Sydney 2037, Australia; 7 Istituto Ganassini S.p.a., Via Carlo Boncompagni, 63, 20139 Milano, Italy

**Keywords:** hyaluronic acid, arginine, cross-linking, bioactivity, cosmeceutical, skin, lung

## Abstract

Hyaluronic acid (HA) is a polymer with unique biological properties that has gained in interest over the years, with applications in pharmaceutical, cosmetic, and biomedical fields; however, its widespread use has been limited by its short half-life. Therefore, a new cross-linked hyaluronic acid was designed and characterized using a natural and safe cross-linking agent, such as arginine methyl ester, which provided improved resistance to enzymatic action, as compared to the corresponding linear polymer. The antibacterial profile of the new derivative was shown to be effective against *S. aureus* and *P. acnes*, making it a promising candidate for use in cosmetic formulations and skin applications. Its effect on *S. pneumoniae*, combined with its excellent tolerability profile on lung cells, also makes this new product suitable for applications involving the respiratory tract.

## 1. Introduction

Recently, requirements related to the biodegradability and the biomimetics of biomaterials have become more stringent for applications in the health and nutritional fields. Biomaterials can be protein- or nucleic acid-based compounds [1,2,3]; however, polysaccharides and the latter are raising increasing interest for medical applications due to their lack of toxicity and physiological inertness. Hyaluronic acid (HA), a biopolymer with hydrophilic characteristics and consisting of repeating units of β-1,4-glucuronic acid and β-1,3-N-acetyl-D-glucosamine, is a typical representative of this class that is well known and widely applied [4].

HA is ubiquitous in the human body, but it is localized in the extracellular matrix (ECM) of connective tissues [5] where it performs both structural functions, derived from its viscoelastic properties, and specific functions, such as the wound repair process, cartilage regeneration, and ocular homeostasis. In humans, the largest amount of HA is present in the skin, which contains nearly 50 percent of the total content [6]. HA is a unique macromolecule used in cosmetic, medical, food, and pharmaceutical products due to its biological and viscoelastic properties.

It has been reported that the administration of high-molecular-weight hyaluronic acid (HMW HA) suppressed acute lung damage by *E. coli* and reduced pulmonary edema formation and alveolar inflammation in human lungs that had been perfused with *Pseudomonas aeruginosa* bacterial pneumonia [7].

It is well known that the HA turnover in vertebrate tissues is rapid because of the degradation processes [8]. In an organism, HA degradation occurs through two main mechanisms: enzymatically by a family of enzymes called hyaluronidases (HYALs) and non-enzymatically, including the degradation reaction by the action of free radicals (ROS) [9,10].

HYALs are the main enzymes responsible for hyaluronic acid degradation: In general, these are endoglycosidases that randomly cleave the β-N-acetyl-D-glucosaminide bonds of the polymer chains leading to smaller oligosaccharides, as well as to the low-molecular-weight HAs that have pro-angiogenic properties [11].

Free radical species such as superoxide, hydrogen peroxide, nitric oxide, and peroxynitrite also degrade HA through oxidation: In the body, these radicals are the result of inflammatory processes, tissue damage, or tumorigenesis [12,13]. Their action is to mediate the hydrolysis of HA by cleaving the glycosidic bonds, thus resulting in HA fragmentation [14].

Temperature and pH values are other factors involved in HA degradation through acid and alkaline hydrolysis as well as thermal degradation. As an indicator of such degradation, viscosity was shown to decrease exponentially as a function of temperature [15]. As a primary constituent of the interstitial barrier, HA degradation reduced its viscosity, which led to an increase in tissue permeability [16].

For this reason, some bacteria, such as *Staphylococcus aureus*, *Streptococcus pneumoniae,* and *Clostridium perfringens*, produce hyaluronidase to increase their mobility through body tissues and as antigenic masking for immune system phagocytes [17,18,19,20]. Similarly, *Propionibacterium acnes*, which is one of the most common skin microorganisms, is known to engage in HA-degrading activity conferred by the enzyme hyaluronate lyase (HYL) [21]. The importance of the aforementioned bacteria as causative agents of infections associated with implanted medical devices is also being increasingly recognized, due to their ability to form biofilms [22]. Different clinical settings have provided evidence for the anti-biofilm effects of HA, and its derivatives, as a non-antibiotic platform with a good safety profile and anti-adhesive properties [23].

In general, to improve the mechanical properties and degradation behaviors of HA, it has been subjected to modifications, and among them, the most popular have been cross-linking strategies by condensation reactions, enzymatic cross-linking, disulfide cross-linking, click chemistry, and polymerization to form double bonds of different chains.

Chemical modifications of HA have also been performed to increase the short half-life of HA solutions after injection into the human body in order to modulate or control the desired therapeutic action. This approach has yielded an insoluble and stable biomaterial that retained the beneficial properties of HA with a longer therapeutic lifetime [24]. Modifications made to HA to achieve better-performing products did not alter its original properties, such as biodegradability and muco-adhesiveness [6,25]. Several in vitro techniques have been reported in the literature that monitored the degradation rates of the properties of cross-linked HA hydrogels, including viscosity change, water content change, and colorimetric assay [26]. The cross-linking of polymers with hydrophilic characteristics, such as HA, has made it possible to obtain elastic and deformable structures called hydrogels. The high degree of swelling that characterizes hydrogels has been shown to be closely related to parameters such as the structure and the degree of cross-linking [27].

To date, HA-based injectable hydrogels have found clinical applications as moisturizers and anti-aging treatments for the skin, as lubricants for joints, in wound healing and tissue regeneration, as anti-inflammatory agents, as cancer therapy, and for the encapsulation and delivery of cells and drugs [28,29,30,31,32,33,34]. Cross-linked photo-thermal antioxidant-injectable hydrogels showed, in vitro, excellent photo-thermal antibacterial properties against *E. coli* and *Staphylococcus aureus*. In vivo studies have also shown that one of these hydrogels significantly eliminated *E. coli* infection, reduced inflammation, and promoted angiogenesis and wound healing in *E. coli* patients [35]. The molecular weight and concentration of HA govern its anti-microbial properties [36].

Among the various approaches that have been used for HA cross-linking, one of the most studied is the modification of accessible hydroxyl groups with homo-bi-functional cross-linking agents, such as 1,4-butanediol diglycidyl ether (BDDE), glutaraldehyde, ethylene sulfide, methacrylic anhydride, and divinyl sulfone (DVS) [37]. These derivatives have been employed for intra-articular and skin-filler injections.

The use of such compounds as cross-linking agents has streamlined the cross-linked HA synthesis and provided good mechanical properties. However, these compounds, once released for HA hydrolysis, have been potential sources of adverse reactions, with many recognized as mutagenic and toxic molecules [38]. However, these compounds are considered acceptable by the FDA because they are present only in trace amounts in their free form and, thus, are considered negligible. At the same time, however, they have been shown to promote inflammation in a concentration-dependent manner [38,39] and are, therefore, as stated above, potentially dangerous when released from their polymeric lattices because of the gel-hydrolysis process.

The capacity for multiple activities is a valued property in drugs, and this has been achieved through the different chemical moieties within a same entity. Furthermore, cross-linked hyaluronic acid has drug-carrier properties due to the formation of cages of different shapes in relation to the degree of cross-linking and the nature of the cross-linker itself [40]. During previous studies, we explored the possibility of obtaining cross-linked hyaluronic acids by forming amide bonds between the carboxyl groups of two different linear hyaluronic acid chains, as well as the possibility of employing it in the delivery of small molecules [40]. More recently, we also explored the possibility of using ornithine as a cross-linking agent in view of its biological properties, obtaining increased resistance to enzymatic degradation and conferring interesting biological properties against lung inflammation [41]. In addition, we recently hypothesized, for the first time, that cross-linked hyaluronic acid could be used in a collyrium preparation, which could be useful in treating eye-dryness syndromes [42,43].

In view of the interesting results obtained, we decided to continue our efforts by studying another amino acid as a cross-linker, namely arginine (Arg), which has never been investigated in this regard. A synthetic strategy was developed that used the methyl ester of Arg through a procedure involving the formation of a triazine derivative with CDMT (2-chloro-4,6-dimethoxy-1,3,5-triazine), which in turn reacted with HA to obtain a cross-linked polymer that showed good bioactivity and mechanical properties within the frame of a safe-by-design approach [44].

Furthermore, Arg is of interest because it is a physiologically active component present in the body and used in nutraceuticals and cosmetics as an active ingredient. It has already been recognized as an anti-aging, immune stimulant and a promoter of tissue repair [45]. In addition, its chemical structure enables it to be used as a cross-linker that features homo-bi-functional amine residues.

Arg is involved in several biological processes. For example, it is the substrate for several reactions leading to the synthesis of other amino acids and acts as a substrate to produce nitric oxide via endothelial cells, thereby regulating vascular tone and, in general, cardiovascular homeostasis [46]. Arg is also involved in angiogenesis and cell proliferation, and it is an indirect precursor of collagen synthesis via the proline pathway [47,48]. Furthermore, Arg is part of the natural hydration factor (NMF) and is considered effective and non-toxic [49]. The use of basic amino acids, such as poly-L-arginine, has been found to be a promising strategy for developing antibacterial agents for the food and cosmetic industries, especially when its activity its enhanced by the carrier [50].

HA and Arg, therefore, have safe profiles associated with their beneficial effects, and for these reasons, they were selected to obtain a new multifunctional cross-linked product with a biological profile that was evaluated as a possible adjuvant for the treatment of pulmonary inflammation and skin disorders.

## 2. Results and Discussion

### 2.1. Synthesis of Cross-Linked HA–Arg

**Figure 1:**
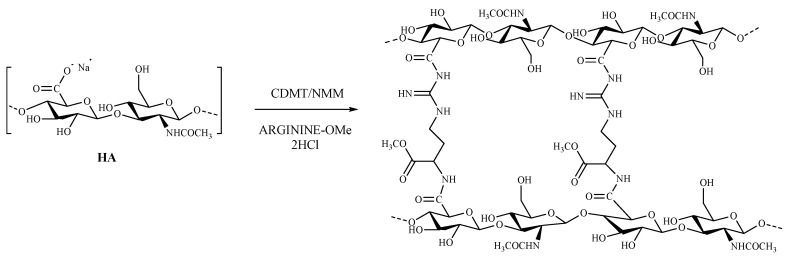
Schematic synthesis of the cross-linked HA-Arg.

The choice of the synthetic procedure followed the criteria of simplicity of handling and the efficacy of the reagents. Finally, we adapted the Bergman method [51], which involved amidation of the carboxyl groups of HA, yielding the desired product by reaction of the amino acid arginine methyl ester to form diamide bonds between the two HA chains. This scaled-up study identified in the association of CDMT with N-methyl morpholine (NMM) as the most efficient activator. The synthesis of HA–Arg was carried out with an excess of both the activating agent and the amino acid (the ratio of HA: CDMT: amino acid 1:3:1.5), at room temperature. For optimal solubilization of the reagents, the reaction was carried out in a deionized water–acetonitrile mixture at a 3:2 ratio. The first step of the reaction involved the activation of the carboxylic acid with CDMT to form the intermediate-activated HA. Subsequently, NMM was added to the mixture to neutralize the chloride ions formed in an equimolar ratio to CDMT (Figure 1).

The resulting hydrogel was subjected to purification by exhaustive dialysis (24 h against water, 48 h against 0.1 M NaCl, and finally, 48 h against water). Finally, the cross-linked hydrogel was lyophilized to obtain a product with a spongy appearance.

### 2.2. Characterization of Cross-Linked HA–Arg

#### 2.2.1. H-NMR

**Figure 2:**
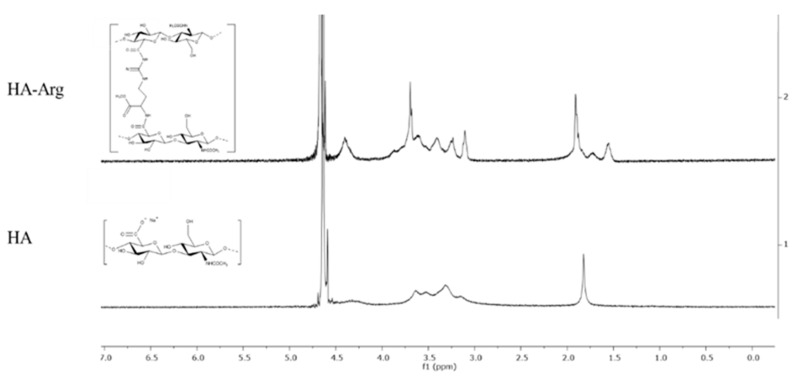
^1^H-NMR profile of cross-linked HA–Arg and native HA.

NMR spectroscopy is the most common tool for the first characterization of the extent of the chemical modification that occurred on native HA. Specifically, the calculation of the integration of a typical signal of the cross-linker (methyl groups on side chains) and not overlapped on the native HA peaks, as compared to that of the characteristic peak of the protons of the methyl group of N-acetyl glucosamine at 1.9 ppm of HA, enabled us to quantify the degree of modification. The new signals detected in the HA–Arg spectrum (Figure S1, Supplementary Information) corresponded to the methyl groups of arginine at position γ (1.55 ppm), β (1.72 ppm), δ (3.10 ppm), and the methoxy group (3.68 ppm) (Figure 2). The multiple signals in the 3.2–3.9 ppm region corresponded to the protons of the HA component. However, HA also showed a large 4.4 ppm signal belonging to the two anomeric protons. In the spectrum of HA–Arg, this signal was superimposed on the signals of the methyl groups in α at 4.4 and 4.5 ppm, respectively. From the calculation of the ratio between the two reference signals mentioned above, it emerged that the degree of modification was estimated at around 40–45% for HA–Arg, a value that corresponded to the quantity of arginine cross-linked with native HA.

#### 2.2.2. Ninhydrin Assay

To further validate the cross-linking efficiency and confirm the absence of traces of unreacted amino acid, the number of free amines was determined on the covalently bonded product HA–Arg and compared with the number of primary amino groups detectable in a reference mixture. This reference consisted of a physical mixture containing an HA–Arg ratio equal to the composition of the bonded Arg suggested by the NMR analysis.

The results obtained from the ninhydrin colorimetric assay (data not shown) confirmed that the Arg–OMe amino groups were covalently linked to the HA with an overall cross-linking efficiency coincident with that estimated by the ^1^H-NMR analysis (43.5%).

#### 2.2.3. Infrared Spectroscopy

The peak positions of characteristic groups of the native HA and the cross-linked HA–Arg are listed in Table 1.

Infrared (IR) spectroscopy is a valuable tool that enabled the study and characterization of natural polymers and is often used in this field to determine the type of bond that is formed following chemical modification.

The IR profile of the native HA was characterized by an absorption band related to the stretching of the N-H and O-H groups at 3267 cm^−^^1^; there were also two bands attributable to the asymmetric and symmetric C=O stretching modes of the planar carboxylate groups (1610 and 1404 cm^−^^1^) and, finally, a band relating to the C-OH stretching vibrations at 1040 cm^−^^1^.

The cross-linking reaction led to different evidences in the HA–Arg spectra (Figure S2, Supplementary Information). The first concerned the appearance of an amide C=O band due to the cross-linking reaction. Specifically, the band at 1636 cm^−^^1^ was the signal that corresponded to the new amide bond formed between the amino acid diamine and the native HA carboxylate group. A second observation was related to the disappearance of the band of the carboxylate groups, which corresponded to an increase in the intensity of the peak at about 3200 cm^−^^1^, relative to the NH group, and the appearance of a peak at 1560 cm^−^^1^, attributable to the NH bending band. Finally, another new band could be observed related to the methyl ester groups of the amino acid at 1729 cm^−^^1^.

Signals belonging to the non-derivatized groups of the native HA were also present in the spectrum of the cross-linking product.

The information obtained from the IR spectra supported the occurrence of the cross-linking process and the formation of the expected bond.

#### 2.2.4. Differential Scanning Calorimetry (DSC)

Chemical cross-linking led to a change of different chemical–physical characteristics, which could then be correlated to different thermal behavior.

**Figure 3:**
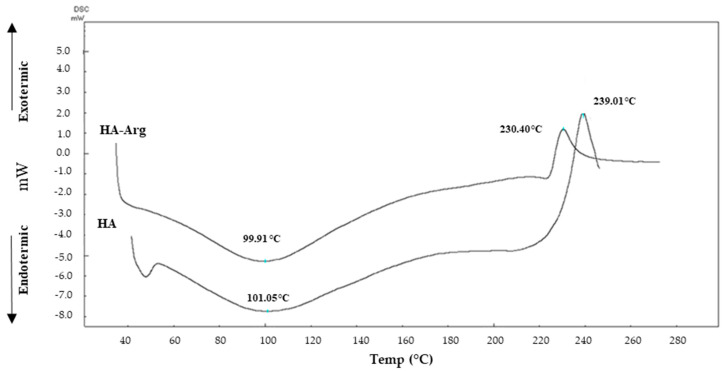
Thermograms of the native HA and HA-Arg.

DSC is a thermal analysis that provides information on hydration and thermal resistance properties [52,53] of polymers. In Figure 3, the resulting thermograms of the native HA and HA–Arg are reported.

The native HA thermal profile showed an endothermic dehydration peak at 101.05 °C, followed by an exothermic decomposition peak at 239.01 °C.

The thermogram of the HA–Arg cross-linking product showed a profile similar to the native HA, with a lower initial temperature. As shown in Figure 3, there was a large endothermic peak at 99.91 °C, which was attributable to the loss of residual moisture after freeze-drying, followed by an exothermic peak of decomposition at 230.40 °C (about 9 °C difference from the native HA). The different values of the thermodynamic parameters obtained for the cross-linked product, as compared to the linear HA (both the endothermic and the exothermic peaks) were attributable to an alteration of the original system and were, therefore, a further confirmation of the presence of a new material that differed from the originator in terms of structural organization.

#### 2.2.5. Scanning Electron Microscopy (SEM)

**Figure 4:**
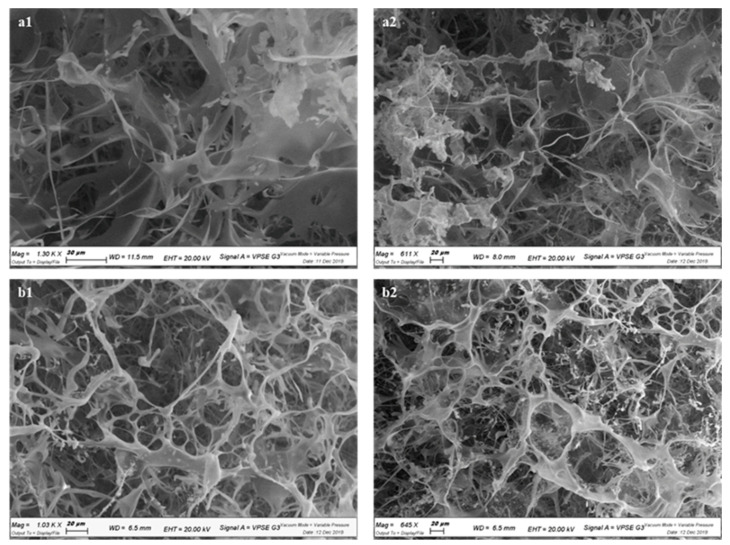
SEM images of HA (panels **a1**, **a2**) and HA–Arg (panels **b1**, **b2**). SEM scale bar: 20 µm (panels **a2**, **b1**, **b2**), 30 µm (panel **a1**).

To observe the morphology of the scaffold, the cross-linked lyophilized hydrogel was studied under a scanning electron microscope (SEM) and compared with the native HA, thus obtaining information regarding the compactness and interconnection of the microstructure (Figure 4).

The images of the native HA (Figure 4, panels a1 and a2) showed a filamentous and irregular structure, while the hydrogel of the cross-linked version had acquired a new structural organization. As shown (Figure 4, panels b1 and b2), a characteristic of the HA–Arg product was the interconnectivity of the circular pores along the entire polymeric matrix that established a solid structure with respect to the linear HA, preventing its rapid dispersion due to hydration in aqueous media. The cross-linked HA–Arg also had a noticeable spherical three-dimensional structure.

#### 2.2.6. Swelling Ratio

The degree of swelling, a gravimetric measure used to study the cross-linking density of a hydrogel, was an essential parameter to evaluate the adhesive and cohesive properties, as well as the drug release. Cross-linking strategies were used to control the rate and the extent of the hydration required to achieve sustained adhesion [54]. As reported in the literature, high swelling values were related to lower cross-linking densities [37].

**Figure 5:**
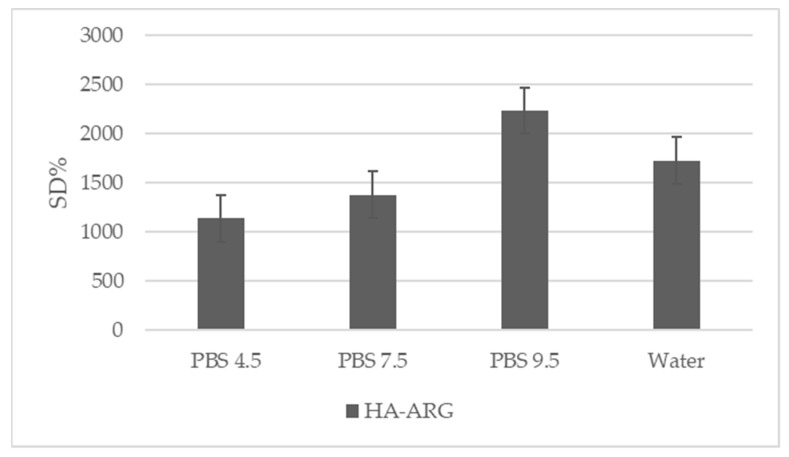
Swelling degree percentage of HA–Arg obtained in PBS at different pH values and in water. Each value is the average of three independent measurements (mean ± StDev).

The swelling values obtained for the cross-linked HA–Arg at 25 °C in water and different physiological pHs [55,56] are shown in Figure 5. It was not possible to measure the swelling of the linear HA, as without cross-linking, it dissolved rapidly when immersed in an aqueous medium.

The results obtained by HA–Arg agreed with the swelling values that are typical of hydrogels, with the swelling property of HA–Arg increasing with the increasing pH.

The highest swelling value was reached in PBS at pH 9.5, probably due to a greater affinity for the basic medium that allowed a greater distension of the derivatized HA chains. It is known [57] that at basic pH values, there is more consistent breakage due to the lower rigidity of the polymeric skeleton, which can be derived from the general hydrolysis of the H-bond network.

The profile obtained indicated that the swelling degree of the cross-linked HA–Arg hydrogel could be modulated by the pH of the medium, and this could be useful for a potential application in the health products field.

#### 2.2.7. Rheology

**Figure 6:**
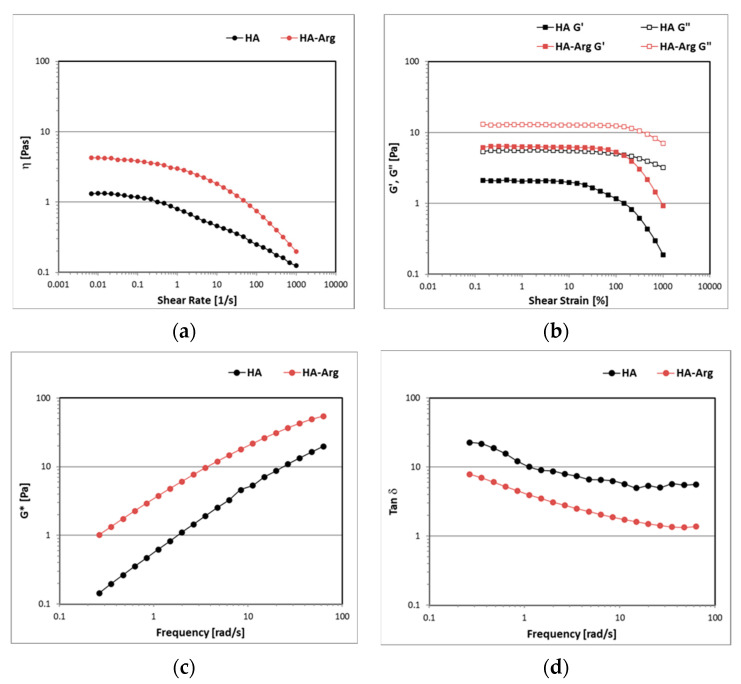
The trend of viscosity values (panel **a**), G′ and G″ moduli (panel **b**), complex moduli (G*) (panel **c**), and damping factor (tan δ) (panel **d**).

The aqueous dispersions of the HA and the HA–Arg at 2% were subjected to a controlled share rate (CSR) rheological analysis in continuous flow conditions in order to study the viscosity as a function of the shear rate, ranging from 0.001 to 1000 s^−^^1^. For both samples, after a Newtonian plateau region at low shear rates, where it remained almost constant, the viscosity progressively decreased as the shear rate increased since the polymeric chains aligned toward the flow direction (Figure 6, panel a). The HA–Arg reached higher viscosity values than the HA due to more robust interactions between the polymeric chains and the solvent, forming a three-dimensional network in which the aqueous solvent became trapped.

The viscoelastic behavior of the samples was investigated under oscillatory flow conditions. the amplitude sweep test enabled the identification of the linear viscoelastic region (LVER) in which the elastic G’ and the viscous G’’ moduli were not dependent on the strain, which showed a consistent trend (Figure 6, panel b). Both the samples showed a liquid-like behavior, with G” always dominating over G’ in the entire range of amplitude strains investigated. The HA–Arg dispersion was characterized by a larger LVER and higher moduli values, in accordance with the results of the continuous flow tests.

Figure 6 (panel c) shows that the G* values for both the HA and the HA–Arg were strongly dependent on the oscillation frequency, as the curves decreased with decreasing values of frequency. As expected, the HA–Arg showed higher values of G* than the HA, as an indication of the higher firmness of the sample prepared with the modified hyaluronic acid. The tanδ values > 1 indicated a liquid-like behavior in which the viscous components always dominated over the elastic ones and no strong bonds were formed (Figure 6, panel d). The HA–Arg showed lower values of tanδ, indicating a higher elastic G’ modulus than the HA and a greater degree of internal structure.

#### 2.2.8. Water Content

The dynamic vapor absorption technique (DVS) enabled us to evaluate the stability of the two polymers, the HA and the HA–Arg, under different relative humidity (RH) conditions (0–90% RH).

**Figure 7:**
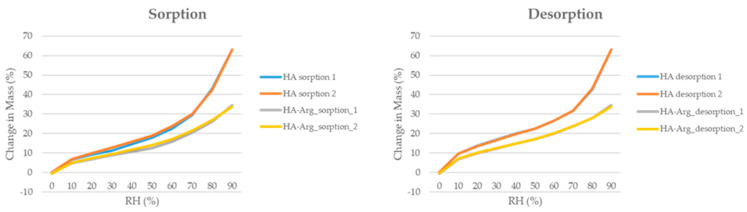
DVS isotherms of two cycles of moisture sorption and desorption of HA and HA-Arg.

Figure 7 shows the resulting absorption–desorption isotherms, confirming the hygroscopic nature of a polymer based on the HA. As previously reported [58], the HA showed a rapid increase in moisture absorption, in the range of 70–90% RH, which correlated to the high capacity of the HA to retain moisture. The HA–Arg profile, instead, showed a slow and gradual increase in the isotherm for the entire range of the RH analyzed.

The absorption curves of the HA–Arg from 0 to 70% were characterized by an increase in the water content, up to about 21%, while at 90% RH, a final water content of about 34% was observed. It is known that the cross-linking process leads to a reduction in the ability of the polymer to absorb water. Therefore, the behavior observed in the HA–Arg agreed with the previous literature [59,60,61].

As for the desorption curves, both the HA and HA–Arg curves were very similar to the absorption curves, with minimal hysteresis phenomena, indicating the process was reversible, excluding permanent changes in the structure.

Therefore, the cross-linking process resulted in a new HA–Arg polymer that retained some of the water-retaining capacity typical of the native HA and, consequently, its properties, as well as less susceptibility in terms of its response to changes in humidity.

#### 2.2.9. In Vitro Degradation

The evaluation of the cross-linking efficiency and the availability of data on the susceptibility to enzymatic degradation of the new cross-linked HA–Arg were fundamental information because they greatly influenced the biodegradability of the hydrogels and the specific fields of application.

Therefore, the susceptibility to in vitro degradation of the samples was performed by hyaluronidase (HAase) to mimic the in vivo conditions, and this was tested using the carbazole colorimetric assay to monitor the D-glucuronic acid content generated during incubation.

The assay was carried out on the cross-linked HA–Arg product, as compared to the native HA and according to the method originally described by Bitter et al. [62].

The degradation was quantified in terms of the increase in the fraction of the soluble sample in the medium in which it had been placed.

The study of enzymatic degradation was comparatively performed on the HA and the cross-linked HA–Arg by bovine testicular hyaluronidase (HAse, 50 U/mL), for up to 24 h.

The method was based on the quantitative colorimetric reaction between the carbazole and the GlcA and involved two consecutive steps: (i) The hydrolysis of the HA supernatant with 0.025 M sodium tetraborate in sulfuric acid to generate the GlcA and the glucosamine monomers; and (ii) the development of the colorimetric reaction between the GlcA and the carbazole.

As shown in Figure S3, the quantity of the glucuronic acid released from the native HA sample was greater than the cross-linked sample for each of the times tested, reached its maximum after 4 h, and then remained almost unchanged. A confirmation of the complete degradation of the sample was also found at a macroscopic level where it was possible to observe a completely clear and homogeneous solution. Complete degradation did not occur, even after 24 h of incubation, for the cross-linked hydrogel. The HA–Arg showed a visually detectable insoluble fraction for up to 24 h with a released GlcA value of 125.1 μg/mL.

After 2 h of degradation, the HA–Arg released a GlcA content equal to 14% (18.51 μg/mL), with respect to the final quantity released, while the native HA released 57.2% (91.42 μg/mL) of the total content. Furthermore, the absence of the plateau suggested that the degradation process did not reach completion after 24 h. The slower release of the GlcA, as compared to the native HA could be attributed to the efficiency of the cross-linking process that had stabilized the HA chains and provided greater resistance to the polymer from enzyme attacks, making it promising for the development of effective delivery systems and viscoelastic applications.

### 2.3. Antimicrobial Activity

**Figure 8:**
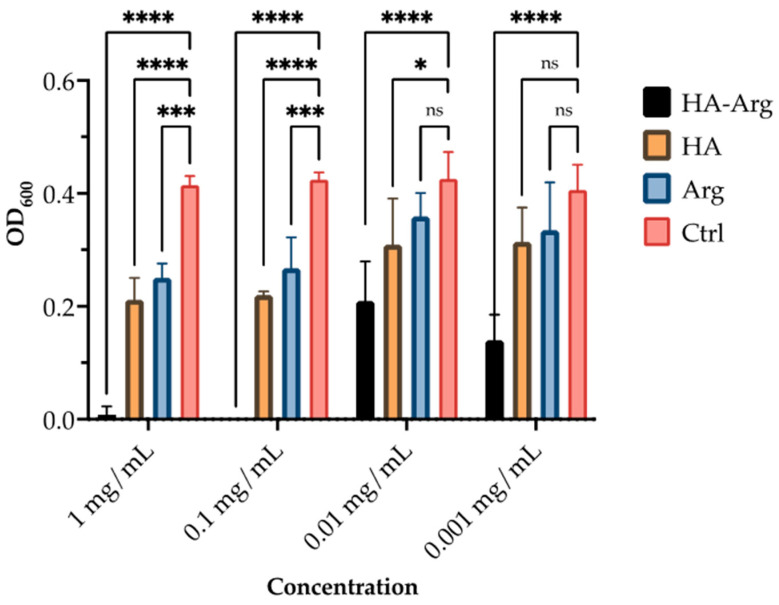
Antimicrobial activity of HA, Arg, and HA–Arg, against *P. acnes*. Bacterial growth was quantified by OD_600_ using a spectrophotometer. Data are the mean of 3 independent experiments performed in triplicate (mean +/− SD); ns *p* ≥ 0.1; * *p* < 0.1; *** *p* < 0.001; **** *p* ≤ 0.0001 (TWO WAY ANOVA followed by Dunnett post test).

**Figure 9:**
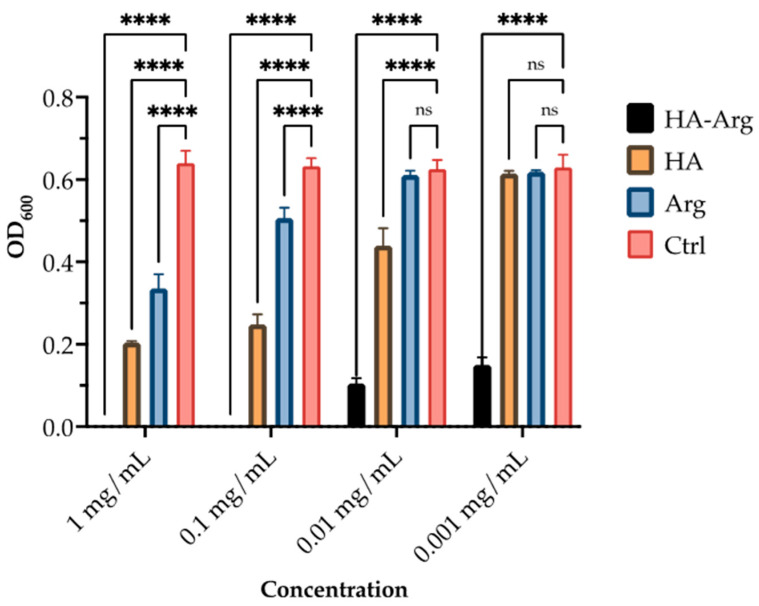
Antimicrobial activity of HA, Arg, and HA–Arg, against *S. aureus*. Bacterial growth was quantified by OD_600_ using a spectrophotometer. Data are the mean of 3 independent experiments performed in triplicate (mean +/− SD); ns *p* ≥ 0.1; **** *p* ≤ 0.0001 (TWO WAY ANOVA followed by Dunnett post test).

**Figure 10:**
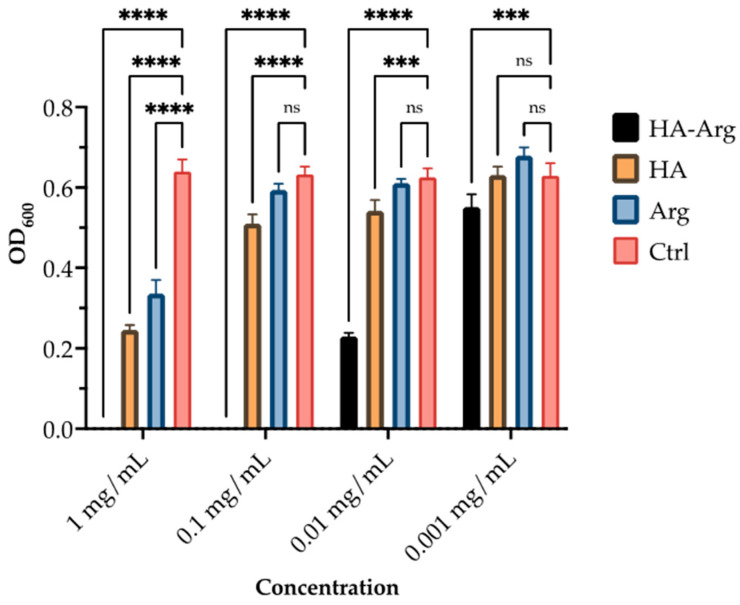
Antimicrobial activity of HA, Arg, and HA–Arg against *S. pneumoniae*. Bacterial growth was quantified by OD_600_ using a spectrophotometer. Data are the mean of 3 independent experiments performed in triplicate (mean +/− SD); ns *p* ≥ 0.1; *** *p* < 0.001; **** *p* ≤ 0.0001 (TWO WAY ANOVA followed by Dunnett post test).

The native HA and HA–Arg were screened in 96-well plates for their in vitro antimicrobial effectiveness. As shown in Figure 8, Figure 9 and Figure 10, the HA–Arg significantly inhibited growth when used up to a concentration of 0.01 mg/mL, both on *S. aureus*, *S. pneumoniae* and *P. acnes*. Its activity was enhanced when used at 0.1 mg/mL and 1 mg/mL, with no bacterial survival detected. All compounds exerted a growth inhibitory effect when used at the 1 mg/mL and 0.1 mg/mL concentrations, as compared to the control (which consisted of untreated bacteria). Considering the activity against *P. acnes* at the 0.1 mg/mL concentration, 3 out of 3 tested molecules inhibited its growth by 42–99%, while only the HA–Arg and, partially, the HA, were able to significantly inhibit its growth when used at 0.01 mg/mL (by 44% and 38%, respectively). When used at 0.001 mg/mL, only the HA–Arg was able to retain its effect, inhibiting the bacterial growth by 77.5%. Considering the activity against *S. aureus*, at the 1 mg/mL concentration, the HA–Arg completely inhibited bacterial growth, while the HA and Arg inhibited its growth by 50%. At the 0.1 mg/mL concentration, all the tested molecules inhibited growth by 36–99%, while only the HA–Arg and, partially, the HA, were able to significantly inhibit growth when used at 0.01 mg/mL (by 87% and 31%, respectively). When used at 0.001 mg/mL, only the HA–Arg was able to retain the effect, inhibiting the bacterial growth by 83%. Considering the activity against *S. pneumoniae*, at the 1 mg/mL concentration, the HA–Arg completely inhibited the bacterial growth, while HA and Arg inhibited its growth by 60% and 48.5%, respectively; at the 0.1 mg/mL concentration, only the HA–Arg was able to inhibit its growth. When used at lower concentrations, ranging from 0.01 to 0.001 mg/mL, the HA–Arg was able to retain its effect only at the 0.01 mg/mL, inhibiting the bacterial growth by 62%.

### 2.4. MTS Cytotoxicity Assay

Two cell lines representative of both the upper and the lower respiratory regions were used to assess the cytotoxicity of the HA and the HA–Arg, and obtain data regarding the safety of using these products for respiratory applications. A metabolic activity assay was used, and the effect on the cells treated with the new product and the HA, at different concentrations (0.009–0.3% of the HA), was compared against cells in which no treatment had been added. The results showed that the cell viability was greater than 80% for all treatments (both the HA and the HA–Arg polymer), up to 0.15% in both cell lines (Table 2). At the highest concentration tested of 0.3% of the polymer, Calu-3 cells, which are representative of the upper respiratory region, showed great tolerance for the treatment for 24 h, with no cytotoxic effects observed. For H441, which are cells representative of the lower region of the lung, the cell viability was slightly lower than 80% for the HA alone, while for HA–Arg polymer cell viability was 87.7 ± 4.8%. Overall, the results showed that the HA–Arg polymer was well tolerated by these cell lines when exposed for 24 h and could be further tested for use in the respiratory region.

## 3. Material and Methods

### 3.1. Materials

Hyaluronic acid (HA) sodium salt isolated from *Streptococcus equi* with an average MW 1.2 MDa was purchased from Caldic (Origgio, Varese, Italy). Arginine methyl ester (H-Arg-OMe·2HCl) was purchased from Bachem. The 2-chloro-1-methylpyridinium N-methylmorpholine (NMM), NaCl, and acetonitrile were purchased from Sigma–Aldrich. The 2-chloro-dimethoxy-1,3,5-triazine (CDMT) was purchased from TCI. The phosphate buffer saline (PBS) at different pH values was purchased from Sigma-Aldrich.

### 3.2. Synthesis of Cross-Linked HA–Arg

A total of 264 mg of sodium hyaluronate (0.66 mmol of carboxylate group) was dissolved in deionized water (52 mL) before the addition of acetonitrile (35 mL). The homogeneous solution was cooled for 0.5 h in an ice bath, and then 347.5 mg of CDMT (1.98 mmol) was added. One hour later, 258.5 mg of Arg-OMe^.^ 2 HCl (0.99 mmol) were added. After adjusting the pH value to 6.5 with 0.5 M NaOH, 217.8 μL of NMM (1.98 mmol) was added, and the reaction was then left under constant stirring (about 400 rpm) overnight. The HA–Arg product was then purified against H_2_O for 24 h, followed by NaCl (0.1 M) for 2 days, and finally against water for 2 days, using a dialysis membrane tube with a Mw cut-off of 3.5 KDa (Thermo Fisher Scientific, Rockford, Illinois, USA); finally, the product was lyophilized using a Lio-5P lyophilizer (Vetrotecnica Srl, Padua, Italy). The final product was obtained as a white spongy material with a yield of 76% (290 mg), and it was kept under refrigeration until use. Note: ^1^H NMR (400 MHz, D_2_O) δ ppm: 4.40 (m, 3H, O–CH–O;—CH α), 3.95–3.10 (m, 15H, C–CH–O; –OCH_3_), 3.107 (m, 2H, CH_2_δ), 1.97 (s, 3H, CO–CH_3_–N), 1.80–1.43 (m, 4H, C–CH_3_–CO and –CH_2_).

### 3.3. Physico–Chemical Characterization of HA–Arg

#### 3.3.1. Ninhydrin Assay

The quantification of free amines was determined using an adapted method by Romberg et al. [63]. A total of 50 μL of HA–Arg sample (0.5% solution in PBS pH 7.4) were combined with 50 μL of ninhydrin reagent (Sigma-Aldrich), consisting of a 2% solution of ninhydrin and hydrindanthin in DMSO and lithium buffer acetate, pH = 5.2. The solution obtained was boiled for 10 min at 100 °C.

After cooling to room temperature, the solution was diluted with 1.5 mL of 50% EtOH solution, and the absorbance was recorded at 570 nm by UV spectroscopy.

The quantification was performed against the Arg-OMe standard calibration curve with the free amino group, in a linear range of 0.1–1.5 mg/mL with R^2^ = 0.9929 ± 0.0015.

#### 3.3.2. Infrared Spectroscopy

Fourier transform infrared spectroscopy analysis was performed on dry samples using an FTIR spectrophotometer PerkinElmer Spectrum 100 with ATR method (attenuated total reflectance) scanned from 4000 to 650 cm^−^^1^, at room temperature. The FTIR spectra were obtained after placing the samples on the crystal, following 8 scans with a resolution of 1 cm^−^^1^.

#### 3.3.3. Thermal Analysis

A thermo-analytical test was carried out on lyophilized HA, and HA–Arg. The thermal transition of the samples was determined by a differential scanning calorimeter (DSC60A, Shimadzu Corporation Kyoto, Japan) calibrated with a pure indium standard. The samples, between 2 and 6 mg, suitably placed in hermetically sealed aluminum pans, were heated from 25 °C to 300 °C at 10 °C/min. Measurements were performed in an atmosphere of purged inert nitrogen at a flow rate of 45.0 mL/min. The TA-60 WS system software (Shimadzu corporation Kyoto, Japan) made it possible to process endothermic and exothermic peaks.

#### 3.3.4. Scanning Electron Microscopy (SEM) Morphological Analysis

The morphology of lyophilized samples HA and HA–Arg was observed using a field emission scanning electron microscope (Zeiss EVO 40XVP, Carl Zeiss Pty Ltd., Oberkochen, Germany), with a voltage of acceleration of 20 kV. The freeze-dried powder samples were deposited on carbon sticky tabs, analyzed in variable pressure mode, and scanned and photographed randomly.

#### 3.3.5. Swelling Degree (SD)

The percentage swelling degree (DS) of HA–Arg was calculated as the ratio between the weight of the swollen gels (Ws) after extensive dialysis in an aqueous medium at different pH values (4.5, 6.5, and 9) with respect to the weight of the gel buckets (Wd). Briefly, exactly weighed 100 mg of dry lyophilized samples were placed in a dialysis tube against 300 mL of PBS at the aforementioned pH values, or in deionized H_2_O at 25 °C. After 24 h, the weight of each swollen sample was determined to calculate the SD according to Equation (1).

% SD = Ws/Wd(1)

#### 3.3.6. Rheology

The rheological tests were performed using a rheometer MCR-101 from Anton-Paar (Anton Paar GmBH, Graz, Austria), equipped with a PP50-P2 sensor, consisting of parallel plates with serrated surfaces, at a fixed gap of 1 mm. The analyses were conducted both under continuous and oscillatory flow conditions at a fixed temperature of 23 ± 0.05 °C and controlled through a Peltier heating system.

The flow curves, which described the trend of the viscosity (η) values as the shear rate increased from 0.001 to 1000 s−1, were determined with a controlled shear rate (CSR) test.

The samples’ viscoelastic behavior was evaluated by analyzing the trend of the storage (G’) and the loss (G’’) moduli through tests under oscillatory flow conditions [64]. An amplitude sweep (AS) test was performed to identify the linear viscoelastic region (LVER), varying the strain (γ) from 0.01 to 1000% at a fixed value of frequency (1 Hz). In this region, both the G’ and the G’’ moduli assumed constant values until reaching a critical strain after which the material was irreversibly modified.

A frequency sweep (FS) test was conducted by decreasing the values of the frequency in a range from 10 to 0.01 Hz, at a fixed percentage value of γ inside the LVER, in which the sample remained unchanged. This test enabled us to characterize the samples’ inner structure and evaluate their physical stability by considering the complex modulus (G*) parameter, defined as the ratio between the shear stress and the shear strain under oscillatory conditions, as well as the damping factor (tan δ), calculated from the ratio between G″ and G′ moduli [65].

#### 3.3.7. Dynamic Vapor Sorption

Using dynamic vapor sorption (DVS, DVS-1, Surface Measurement Systems Ltd., London, UK), the relative moisture sorption of the freeze-dried samples of HA and HA–Arg was analyzed after exposure to different humidity levels (0–90% RH). Each sample, after being placed in an aluminum pan, was exposed to two cycles of 0–90% relative humidity (RH) at 25 °C, with 10% RH increments. The equilibrium moisture content in each moisture phase was determined as a change in the ratio of mass-to-time (dm/dt) of 0.0005% min^−^^1^.

### 3.4. In Vitro Enzymatic Degradation Assay

A total of 1 mL of an aqueous solution (1.5% *w*/*v*) of HA and HA–Arg, respectively, was freeze-dried after being poured into spherical molds with a diameter of 10 mm. The freeze-dried samples were then pressed to obtain compact solid discs with a thickness of 1 mm and a weight of 15 mg.

The discs of each sample were placed in a stock solution of hyaluronidase (HAse) from bovine testes (type IV-S powder, 1045 units/mg, lot SLCC9109, from Sigma Aldrich) and prepared at a concentration of 50 units/mL in PBS. To check the quantity of glucuronic acid released, the samples were kept under stirring at 37 °C for the various test times (up to 24 h). The absence of temperature-related degradation phenomena was verified by carrying out a control test for each sample in the absence of HAse at the same temperature (data not shown).

Briefly, 200 μL of supernatant was withdrawn at each predetermined time and added to 3 mL of 0.025 M sodium tetraborate in sulfuric acid. The samples were then vortexed, boiled for 10 min at 100 °C, cooled, and added, with 100 μL of carbazole reagent, at 0.0125% in EtOH. The reaction was then started by boiling the solution for a further 15 min. By reading the absorbance at 523 nm using UV-31 WAVE SCAN (Giorgio Bormac spectrophotometer Srl, Carpi (MO), Italy), we monitored the amount of GLCA produced after degradation. A blank control was prepared with phosphate buffer only.

### 3.5. Biological Activity

#### 3.5.1. Assessment of the Minimal Inhibitory Concentration (MIC) of the Compounds against *Staphylococcus aureus* and *Propionibacterium acne*

A loop of a single colony grown on tryptic soy agar (TSA) for *Staphylococcus aureus* and *Streptococcus pneumoniae* (*S. aureus* and *S. pneumoniae*) and in Columbia agar sangue (ColA) for *Propionibacterium acnes* (*P. acnes*) was transferred to liquid tryptic soy broth (TSB), *S. aureus* and *S. pneumoniae*, and TSB supplemented with brain–heart infusion (BHI), *P. acnes*, and incubated at 37 °C for 24 h (*P. acnes* was incubated under anaerobic conditions for 48 h). Subsequently, after reading bacterial density with a spectrophotometer, the suspension was diluted in TSB (and TSB + BHI) to reach the density of 1 × 10^5^ cells/mL. Afterwards, serial dilutions of each compound were performed in TSB (and TSB + BHI) in a 96-well plate (Corning). Then, 100 μL of the previously prepared microbial suspension was added to each well. The highest concentration of each compound tested in this experimental setting was 1 mg/mL, and it was added ×10 in each subsequent well of the 96-well plate, up to 1 ng/mL. Plates were incubated for 24 h and up to 5 days, at 37 °C, under constant shaking (*P. acnes* plates were incubated under anaerobic conditions). After incubation, the turbidity of the bacterial culture was measured using a spectrophotometer microplate reader (Spectramax Tecan-Fluoroscan, Tecan Italia, Cernusco sul Naviglio, MI, Italy) with a wavelength of 600 nm. A microbial culture, in which no compounds had been added, served as a growth control.

#### 3.5.2. Cells Culture

Cytotoxicity was assessed in two lung cell lines, Calu-3 and NCI-H441 (carcinoma-derived epithelia, ACTT, Rockville, MD, USA), derived from bronchial and alveolar regions, respectively. Calu-3 was cultured in Dulbecco’s Modified Eagle’s Medium/F-12, supplemented with 1% (*v*/*v*) non-essential amino acids, 1% (*v*/*v*) 200 mM L-glutamine solution, and 10% (*v*/*v*) fetal bovine serum (FBS), while H441 cells were cultured in RPMI-1640 medium, supplemented with 10% (*v*/*v*) FBS. Media were changed 2–3 times per week until confluency (70–80%); then, cells were passaged using trypsin at 1:3 and 1:7 ratios for Calu-3 and H441 cells, respectively. Both cell lines were cultured in 95% humidified air and 5% CO_2_ atmosphere, at 37 °C.

#### 3.5.3. MTS Assay

Cells were seeded at a density of 5 × 10^4^ cells/well in a 96-well plate. After 24 h, the media were removed, and 100 µL of the raw materials prepared in HBSS was added to the wells. Cytotoxicity was assessed at 24 h after treatment via measurement of metabolic activity of cells exposed to treatment, as compared to control (no treatment-diluent only). In the assay, 20 µL of MTS reagent was added to the wells and read after 3 h at 490 nm. The solutions were prepared as a serial dilution in the range of 0.009–0.30% (*w*/*v*) for HA and HA–Arg. DMSO 20% was used as the control for cell death.

### 3.6. Statistical Analysis

All values were the result of at least three separate determinants and were reported as mean ± standard deviation. The statistical software GraphPad Prism (version 8.2.1) was used to determine the one-way and two-way ANOVAs in order to verify the significance of each experiment. Significance was determined as *p* < 0.05.

## 4. Conclusions

The aim of this work was to develop a new cross-linked hyaluronic acid, according to a safe-by-design approach and characterized by lack of toxicity on lung cells and resistant to enzymatic degradation. A new synthetic approach was devised using CDMT as the activating agent for the amino acid arginine methyl ester, which was selected as the cross-linking agent.

The results obtained from the physicochemical investigations confirmed the cross-linking with a significant degree of modification, as proven by the NMR analysis and typical amide C=O infrared stretching. The HA–Arg showed a characteristic thermal behavior of the HA-based polysaccharides with endothermic and exothermic peaks typical of a new polymeric structure.

The rheological analysis showed a liquid-like viscoelastic behavior for the HA–Arg, since G’’ always dominated over G’ in the entire frequency range investigated, with a swelling ratio that increased with increasing pH, making the HA–Arg a new polymer for drug delivery, cosmeceutical, and nutraceutical applications.

The antibacterial properties of the HA–Arg represented an added value for this material, specifically its activity against *S. aureus* and *P. acnes* could be promising for its use in cosmetic formulations for skin applications, and its effects on *S. pneumoniae* may represent a useful application for bronco-pulmonary administration.

## 5. Patents

This work was the object of the University of Ferrara patent application N° 102019000024117, now EP4077403, licensed to Istituto Ganassini Spa.

## Figures and Tables

**Table 1:** Position of main bonds (cm^−1^) in IR spectra of hyaluronic acid before and after cross-linking.

Specimen	Wavenumber [cm^−1^]	Functional Group
HA	3267.14	O-H; N-H
	1610.95	C=O
	1404.00	C=O
	1040.00	C-OH
HA–Arg	3298.94	N-H
	1729.51	COOCH_3_
	1636.04	C=O amide
	1560.15	N-H
	1374.77	C=O
	1038.54	C-OH

**Table 2:** Cytotoxicity of HA and HA–Arg on Calu-3 and H441 cell lines exposed to treatment for 24 h, expressed as mean cell viability ± Stdev (*n* = 3).

Treatments	Calu-3 Cells						H441					
	HA			HA–Arg			HA			HA–Arg		
	Mean		Stdev	Mean		Stdev	Mean		Stdev	Mean		Stdev
Control Media	100.0	±	6.7	100.0	±	0.8	100.0	±	2.0	100.0	±	5.9
HA 0.30%	97.8	±	2.2	100.3	±	3.2	78.7	±	1.0	87.7	±	4.8
HA 0.15%	97.0	±	4.3	96.5	±	6.0	84.2	±	2.3	99.0	±	3.3
HA 0.075%	96.3	±	5.9	98.1	±	0.9	86.5	±	2.5	95.5	±	4.0
HA 0.0375%	100.1	±	2.6	97.9	±	6.7	91.8	±	2.0	98.4	±	1.0
HA 0.018%	93.7	±	3.1	95.6	±	4.3	90.8	±	6.7	112.6	±	5.2
HA 0.009%	93.1	±	5.9	98.0	±	5.6	90.6	±	2.8	105.0	±	4.1
DMSO 20%	47.47	±	1.36	51.99	±	1.78	11.84	±	2.20	14.97	±	1.47

Control media indicates negative control; DMSO 20% is the positive cell death control.

## Data Availability

Data is contained within the article and supplementary materials.

## Supplementary Materials

The following supporting information can be downloaded at: https://www.mdpi.com/article/10.3390/ph16030431/s1, Figure S1: ^1^H NMR spectra of cross-linked product HA–Arg. Figure S2: IR spectra of the native HA and the HA–Arg. Figure S3: Glucuronic acid released from in vitro degradation of the HA and the HA-Orn in PBS, pH 7.4 at 37 °C in the presence of 50U/mL of hyaluronidase.
