# Supplementary material for: A Safe-by-Design Approach for the Synthesis of a Novel Cross-Linked Hyaluronic Acid with Improved Biological and Physical Properties
Source: Pharmaceuticals (Basel). 2023 Mar 11;16(3):431. doi: 10.3390/ph16030431 (PMC10058433; doi:10.3390/ph16030431)
Supplement: Supplementary file 1 [file pharmaceuticals-16-00431-s001.zip › pharmaceuticals-2264428-supplementary.pdf]

A Safe-by-Design Approach for the Synthesis of a Novel Cross-linked Hyaluronic Acid with Improved Biological and Physical Properties

Sabrina Sciabica [1,†], Riccardo Barbari [1,†], Riccardo Fontana [2], Giovanni Tafuro [3], Alessandra Semenzato [4], Daniela Traini [5,6], Dina M. Silva [6], Larissa Gomes Dos Reis [6], Luisa Canilli [7], Massimo Terno [7], Peggy Marconi [2], Anna Baldisserotto [1,*], Silvia Vertuani [1,*] and Stefano Manfredini [1]

[1] Department of Life Sciences and Biotechnology, University of Ferrara, via L. Borsari 46, 44121 Ferrara, Italy
[2] Department of Chemical, Pharmaceutical and Agricultural Sciences, University of Ferrara, via Fossato di Mortara 64/B, 44121 Ferrara, Italy
[3] Unired Srl, via Niccolò Tommaseo 69, 35131 Padova, Italy
[4] Department of Pharmaceutical and Pharmacological Sciences, University of Padova, via Marzolo 5, 35131 Padova, Italy
[5] Macquarie Medical School, Faculty of Medicine, Health & Human Sciences, Macquarie University, Campus Macquarie Park, Sydney 2109, Australia
[6] Woolcock Institute of Medical Research, 431 Glebe Point Road, Glebe, Sydney 2037, Australia
[7] Istituto Ganassini S.p.a., Via Carlo Boncompagni, 63, 20139 Milano, Italy
* Correspondence: bldnna@unife.it (A.B.); vrs@unife.it (S.V.); Tel.: +39-0532-455258 (A.B.); +39-0532-455294 (S.V.)
† These authors contributed equally to this work.

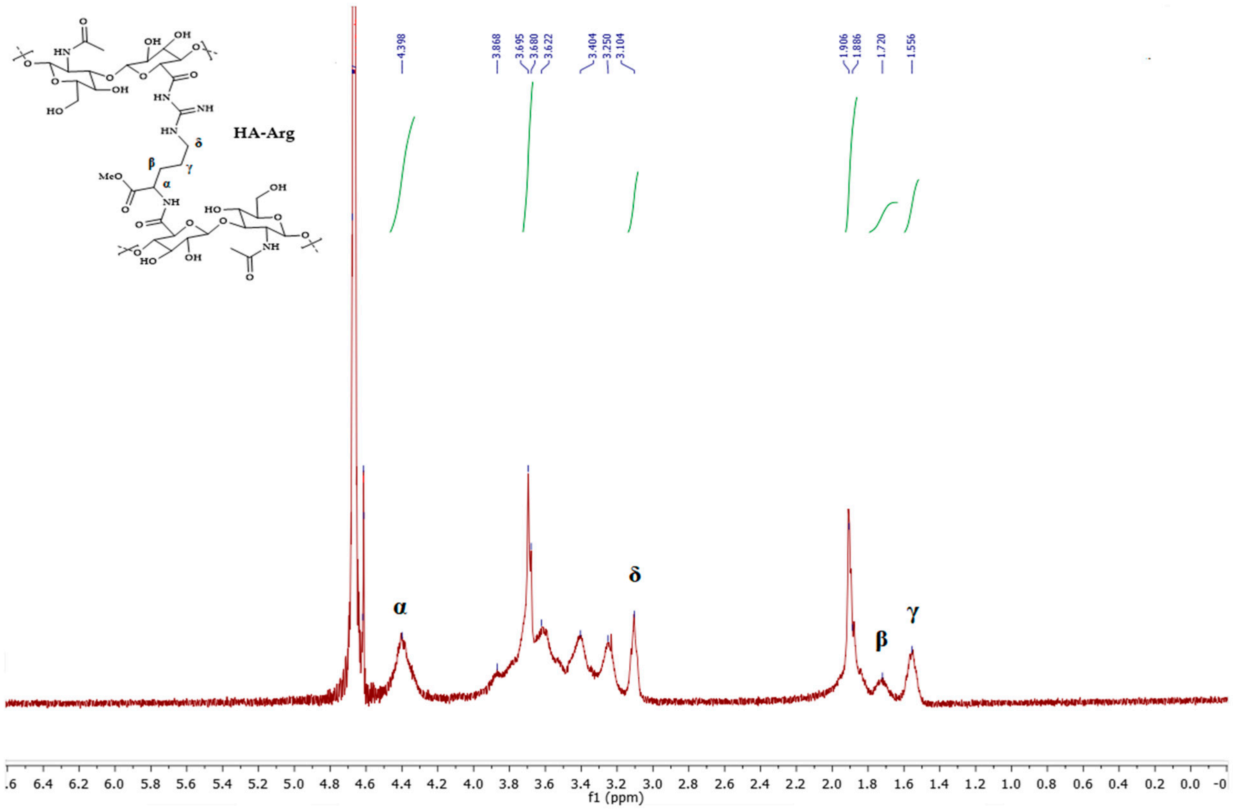

Figure S1. ^{1}H NMR spectra of cross-linked product HA-Arg.

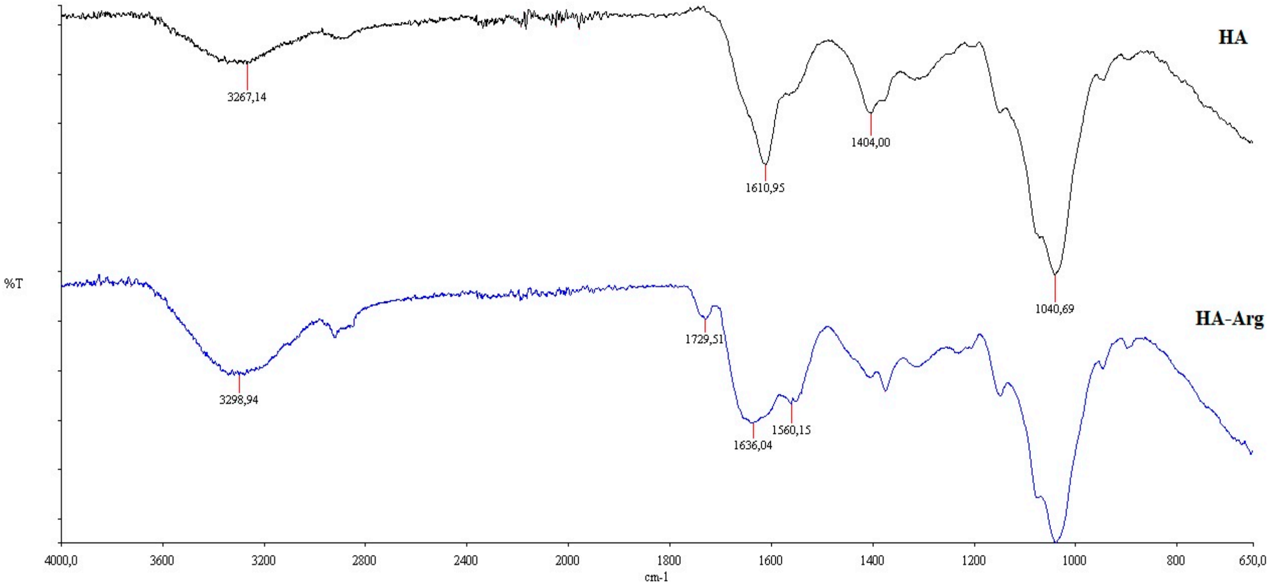

Figure S2. IR spectra of native HA and HA-Arg.

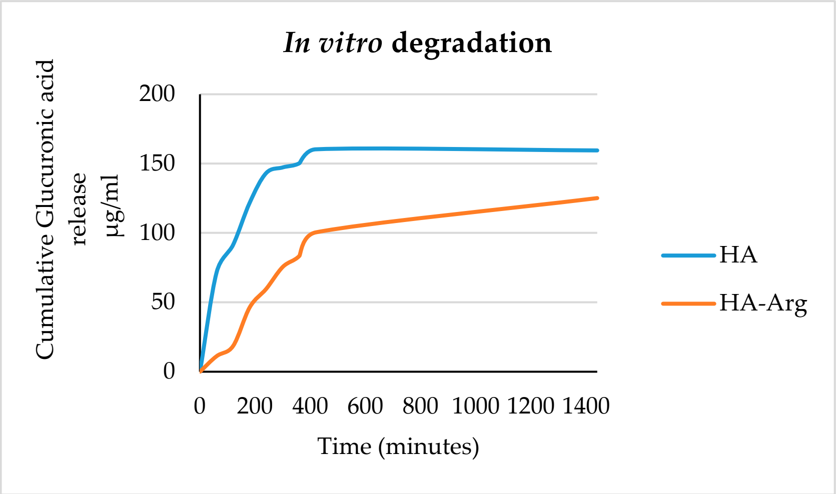

Figure S3. Glucuronic acid released from in vitro degradation of HA, and HA-Arg in PBS, pH 7.4 at 37 °C, with 50U/ml of hyaluronidase.
